# Review of Dr. Leslie's Report of the Proceedings of the Last Meeting of the Mississippi Valley Association of Dental Surgeons

**Published:** 1852-04

**Authors:** James Taylor

**Affiliations:** Cincinnati.


### ARTICLE IV.

*Review of Dr. Leslie's Report of the Proceedings of the last Meeting of the Mississippi Valley Association of Dental Surgeons.* By JAMES TAYLOR, M. D., D. D. S. Cincinnati.

MESSRS EDITORS:

IN the January No. of your valuable Journal, we see a very lengthy article, purporting to be from one Allen Leslie, giving, as he says, a report of the discussions and proceedings of the Mississippi Valley Association of Dental Surgeons, at their last annual meeting.

As we have no knowledge of any individual of that name, we refer to the initials at the close, which are more significant, and enables us to determine with some degree of accuracy, who the author is. A. M., we believe, stands for Master of *Arts*, and L. for ——. We of Cincinnati, and especially of the society, know the application of the initials.

As the "report" does great injustice to the association, the proceedings of which your reporter has trimmed, garbled and distorted, to suit his own views and purposes, and as it has been sent to the Journal "that it may have a wide circulation," we feel it a duty we owe the association, to correct some of the gross misrepresentations with which it abounds, "so that the profession may know *through the pages of the Journal*, what we are *doing* and *saying* out here in the west." To review the entire "report" in detail, would take up more space in your Journal than would be profitable or perhaps interesting to your readers; we shall, therefore, not pretend critically to examine the gentleman's elaborately written "report," and speech or speeches. The first appears to be a very nice little "scientific" affair of only five or six pages, but the second shows much more bottom, for it fills some 20 pages of the "report," and indeed has a *tail* attached which covers some two or three more.

We feel assured that the members of the association will be much amused and edified with the splendid exhibition of talent the gentleman has given us. We were not aware, before, that any thing so exceedingly rich and eloquent had come off at our recent meeting. We might suppose that the most of this occurred during our absence from the hall, and that the president was taking his afternoon siesta in the chair, for he assures us that *much* of the "report" is news to him: but if we had been absent, we could not have taken the personal and active part in the drama assigned us by your reporter. You can, therefore, appreciate the very embarrassing circumstances under which we labor in its examination. First, it appears that we expressed ourself as unable to explain, on "scientific principles, the mode upon which the partial vacuum in the chamber is obtained." "We would not give a full explanation of the *thing*." We believe we did express a doubt whether we should be able to enlighten the learned gentleman on this subject. We still think as we did then, that a perception so astute as to see no passage for air through a hole made in wax or plaster, to the arch of the mouth, would be difficult to enlighten on this or any other subject of the like character. The passage of air was not expected to "immediately" loosen the impression. If there should be a vacuum in the impression at the center of the arch filled with "water or air," this would escape, so says your reporter. Truly, here is a discovery, and it is given with most commendable liberality. The air can get in around the border, and yet to get in at these little holes in the center, is most astonishing, and more than your reporter "can understand." Is it any wonder that we should feel unable to explain the "*thing*" on "scientific principles." We are glad, however, that your reporter has done this "on scientific principles," and trust your readers will not fail to profit by it. We, however, doubt very much whether a majority of our patients would be content with a plate which "but little more than held the weight of the operation and the pressure exerted by the tongue in articulation."

We next find ourself offering to bet, or forfeit, fifty dollars on the success of a first impression with plaster. We believe we did speak quite positive in relation to the success which generally attends our impressions in plaster; but as we universally refuse to bet, we have no knowledge of tempting your reporter in this way, as we failed to enlighten your reporter upon our method of taking impressions at that time; we would state that the plaster carried to the roof of the palital arch on a spatula, though thicker than that which we have in the cup for the impression; requires but little pressure to adapt it to this part of the mouth; far less, indeed, than wax, which can be successfully used. We do not always use salt with the water in mixing the plaster, but only when it does not readily set without it. In this connection, we find our friend Dr. Goddard called up before he had his lesson, he therefore "did not know anything about it," we hope he will be better prepared next time.

We pass on now to that which has engaged the especial attention of your reporter, and fixed his indignant eloquence—that is, the resolution offered to award to Dr. Allen, a gold medal. Here he labors to make an entirely false impression, as that, the president "undertook immediately to take the vote," and thrust it through without an opportunity for discussion, which would have been accomplished if it had not been for the noble daring of one member (the "reporter.") This we say is not true in either respect. The president, as in all cases, called the attention of the society to the resolution, and your reporter made due haste to be first on the floor to discuss the question, and *who* of your readers would doubt his having held it more than all the rest; your reporter has apparently a great fancy for the word, "immediately." The impression is to loosen "immediately." "The president "immediately" proceeds to take the vote, and we say your reporter "immediately" takes the floor, but finding himself unlike the greyhounds, ready for a hot pursuit on an empty stomach, "immediately" moves an adjournment until after dinner.

There was nothing which occurred either before or after dinner which could, by an unprejudiced individual, be construed to warrant the supposition that the resolution, as proposed, would be hastily, or even at all, forced through the society. The manner in which it was seconded showed a different spirit, as it was done with this remark, that "we would second the resolution to bring it before the society," meaning, of course, Dr. Allen's mode of fastening artificial teeth to plates, &c. After dinner we wished to offer a different resolution as a substitute for the one offered by Dr. Goddard. To dispose of that we offered to withdraw our second. The chair and all the members present except your reporter assented to this. Either ourself or the president suggested that courtesy to Dr. Goddard required that we should wait until his return. After his return, the subject (Dr. Allen's improvement) was referred to a committee of three, consisting of Drs. Goddard, Taylor and Leslie; a meeting was appointed for the committee to confer together and prepare a report to be submitted to the society. The committee met in the evening, examined the specimens, and applied the tests, in which they were to lay till morning. The committee was then to meet again and prepare their report before the society convened. This meeting, your reporter did not attend, but prepared a report for himself which was never shown to the balance of the committee. The other members prepared a report which was submitted to your reporter the first opportunity. They reported that they had subjected specimens of work put up by Dr. Allen to satisfactory tests, as to its strength and capability of resisting acids, &c., &c., and thought it possessed peculiar advantages over the ordinary method of setting teeth, and recommended the following resolution:

*Resolved*, That Dr. Allen deserves all commendation for his indefatigable exertions in thus developing and making available, a new and important improvement in mechanical dentistry, and that we recommend this improvement to the profession as worthy of their attention.

Now, what is the just inference from this as to the view the society took of Dr. Goddard's resolution? evidently, that the society was opposed to it, nor was this opposition brought about by the eloquent discussion of your reporter, because cause he had not yet *delivered* himself—only the first symptoms of labor had manifested themselves, none of those violent *throes* and *struggles* which characterized the future progress of the laborer had made their appearance. The attending physicians had as yet no just grounds of anticipating a prolonged and difficult accouchement, hence, ergot, blood-letting nor any such remedies had been thought of as necessary for the case. Only the usual number of sheets had been provided as no *flooding* was then anticipated. The future development of the case shows the importance of proper prognosis and early attention to *bad symptoms.* We presume if this had been attended to aright in this case, the violent *afterpains* might have been prevented. Let it be borne in mind, that the above report and resolution furnished the occasion of your reporter's wonderful onslaught. The subject of patents was not at any time properly before the society, and your reporter was several times called to order by the president during the speech he *did* make as discussing a subject not before us; your reporter says that the "necessity for his speech only became apparent a short time previous to the "this morning's meeting," his report and speech must therefore have been prepared during our walk of some three or four squares, from Dr. Goddard's to the hall in which the society met; let this be, however, as it may, the society met in September and January—we have the full grown *genius* a time long enough for *delivery* if not for *conception* and *gestation.*

We have first an array of extracts in relation to the subject of patents, and our remarks are quoted much as an infidel would quote scripture. It appears that on a former occasion we expressed ourselves against patents, except, indeed, as applied to the mechanical arts, that while "a piece of mechanism might thus with propriety be patented, yet a principle of that science which claims for its object the amelioration of the ills of life, should not be thus trammeled." The sentiment, however, was more fully expressed by the remark "that no agent for the cure of disease should be thus trammeled." We know not that our present sentiments could be more fully expressed than in the above remarks, and there is scarcely room for a doubt as to our practice; yet, your reporter so strenuously urged this as fully committing us against all patents, that it was necessary at the time to correct him.

The resolutions offered by our brother, Dr. E. Taylor, at the second annual meeting of the association, did not entirely meet our views, hence the qualified remarks we then made, and which your reporter construes very different from their true meaning. The second resolution offered by Dr. E. Taylor at this meeting, postponed further action on this subject until the next annual meeting, as we felt at that time not prepared to go the length which some felt it necessary in this matter. The subject was not brought up at our next annual meeting, and, indeed, has never been disturbed since, unless when your reporter disentombed the monster and roused the association from its long and dangerous slumber. Your reporter was not then a member of this association, this may account for his indiscretion in arousing such a dangerous animal. About this time, we are told he was very familiar with his lordship, thought him very beautiful, and even visited his great cage at Washington city, and offered an oblation at his shrine, which, however, did not prove very palatable to his epicurean taste. The reader may learn more of the *style* of this *dish* before we have done with the "report."

The subject of patents has ever been a great *bugbear* to the medical profession; we think this is owing to an imperfect understanding of the subject, identifying it with quackery, charlatanism, &c., &c. We propose, therefore, taking a hasty view of this subject. We have, first, a law of congress, which gives encouragement and protection to the patentee. This law appears to have been especially intended to foster and encourage a spirit of improvement among manufacturers in the mechanic arts, yet embracing in its ample folds science in every department. Are we prepared to set this law at defiance, by thrusting from us those who seek its protection? Are we prepared to throw aside every thing in the shape of an improvement in the arts, which bears the impress of a patent?

What is the difference between a patent and a copy-right? Is the obtaining of a patent, quackery? These questions deserve some notice, and we shall notice them briefly, as proposed.

In relation to the first question, we would remark, that all the laws of the land should receive our support, unless they should cause us to violate a moral obligation or law, which has a higher claim to our observance. The medical profession, regarding the cure of disease as of the first consequence, hence, in assuming the duties of the profession, they impose upon themselves a moral obligation, which shall consult first the preservation of life, and it has thus become a settled principle in medical ethics, that all knowledge essential to this great object, should be free. That all remedies for the cure of disease, should become the property of the profession. The claims of humanity here step in, and demand of the high minded professional man, a sacrifice of what the law recognizes as his own property. This, for the good of the public, and the honor of the profession, of which he is a member, he cheerfully does. This compliance constitutes one of the noblest traits of the medical profession, of which we consider dental surgery a part and parcel. Has compliance, however, generally extended farther than this, and should we demand more? If so, we must at once blot out the record of every patent. We do not believe the public, or the profession themselves, are ready for this. We do not believe that the intelligence of the nation is prepared for it. The medical profession, as a general rule, have not done this. If any of them make a discovery or invention in science or art, the patenting of which cannot, in any way, effect the general and successful treatment of disease, they hesitate not to avail themselves of its benefits. And why should they? They have rights in common with every other good citizen, and none pretend to say that he shall not avail himself of them. We are satisfied the profession would be content if no departure was ever made from this rule. We doubt not, the records of the patent office would show a full proportion of patents, as belonging to the medical profession. We would that none of them were affixed to remedies for the treatment of disease, and notwithstanding the reproach thrown out by some in the medical profession, in regard to dental patents, yet dental surgery aside from mechanical dentistry, will compare favorably in this respect with any other department of the healing art. We scarce have a patent medicine in dental practice, and in mechanical dentistry we would fail to compete with general surgery. We do not allude to this, out of any invidious feeling, nor to, in the least, extenuate the patenting of remedial agents, but to show that the medical profession stands in the same predicament as the dental, and is not prepared to cast the "first stone." And it is, perhaps, far more reprehensible, for in dentistry, this only effects the substitution of an organ which disease has already destroyed, while in the other, it often applies to a mechanical appliance which is designed to preserve an organ, or perhaps, life itself.

The question comes to simply this: is the obtaining of a patent for any thing, justifiable? If so, where is the line to be drawn? If we confine it to manufactures and mechanic arts, it is still not excluded from medical science, for this, in her efforts to combat disease, lays her contributions on science and art. She grasps every resource which an all-wise Providence has opened to her persevering industry.

We are aware, that although the medical profession have generally reprobated the use of patent remedies for the cure of disease, yet this has not so generally been applied to those mechanical appliances which art has contributed for the use of the surgeon. It may be, that too much latitude has here been allowed. They have, at least, set the dental profession an example of charity and forbearance; and as *they* have not thus seen fit to thrust the patentee in the mechanic arts from them, *we* scarcely feel it our duty to set *them* the example. Are we prepared to throw aside every thing in the shape of an improvement in the arts, which bears the impress of a patent?

Our first duty is to consult the greatest good of our patients, for this we strive to perfect ourselves in every department of our profession, and he who brings not into requisition all which is available in our science for the good of his patients, has but a poor appreciation of the duties of a dental practitioner.

This principle, which urges us to seek the greatest good of our patients, is the very foundation of excellence in the profession. We therefore would not throw aside the patent article because it was *patented*, but use it, and if possible, improve it.

In mechanical dentistry, we feel that no patent which lays not a prohibitory duty upon us, shall keep us from every improvement which has or may be made in this department. Thus, if an easier and better method of inserting teeth is devised, we shall adopt it as soon as we can. We do not believe the old way in every thing is the best. We wish to try every thing which holds out good prospects of improvement, and hold on to that which in our hands proves best.

What is the difference between a patent and copy-right?

A patent right makes the discovery or improvement of an individual, his property, and protects him in the use of it. It makes, indeed, a species of property out of knowledge. It secures to the patentee, for a limited time, the product of his mental labor or mechanical skill. As medical men, if this throws no obstacle in the way for the successful treatment of disease, no objection can be urged.

A copy-right secures to an individual a right of property in his knowledge. It makes, as in the other case, knowledge a species of property, and the whole object of the law of copy-right, is to secure to the author* the use and benefit of his knowledge. In both cases, this is really the product of labor, both mental and physical. And the only difference we see in the two laws is, that the law of patents is made to secure to the inventor for a limited time, the practical application of his knowledge, where it presents itself in such form as not to be available by copy-right.

In both cases, the public is taxed, securing to the author or inventor, a remuneration for his labor. If we were to decide on the amount of pecuniary benefit, we should, as a general

* We use the term author, in contradistinction to that of patentee. The former, being applied to written productions, secured by copy-right. The latter, to mechanical improvements or productions, secured by patent right. Both are truly *authors.*

rule, accord to the author who secures himself by copy-right, a much larger remuneration than the patentee obtains. We think there are but few patent rights which pay as well as Macauley's copy-right of the History of England.

We know there is here a distinction which has often been urged, which is, that the publication of an author becomes, so far as knowledge is concerned, public property. That all which is available for public use, is soon disseminated through various channels. This, to a certain extent, is true, and so far, an author claims more liberality than a patentee; yet, is this not more in appearance than reality? With all this liberality, we doubt whether he would suffer his works to be republished by any one, with impunity. He suffers quotations and notices to be made through the public prints; and why? because that which he has for sale, is thus introduced to public notice, and he expects to be benefited thereby. The patentee does the same, and you may even minutely describe his article, tell how it is made and used, and he cares not; when patented, it is no longer a secret. The author and he have both parted with their secrets.

Is the patenting of an article quackery? if so, it is sanctioned by the highest authority of the land. It receives its sanction not merely from a state authority, but has been considered of such importance that congress has reserved this privilege, and, if we mistake not, all civilized nations that have any pretensions to intelligence and progress, have their patent laws sanctioned by the highest authority. If, therefore, it is quackery, it claims an honorable paternity. But we regard it not as quackery or charlatanism, and we refer your reporter to the following resolutions of our society, which *was passed* at its second annual meeting.

Offered by Dr. E. Taylor:

"*Resolved*, That any member of this society who shall extol his own *peculiar* merits over those of a fellow practitioner, or offer his services at lower rates than is common among the members of the profession, among whom he operates, through the public prints, or use any secret nostrum, (unless pledged,) prior to the present time to maintain secrecy, shall be liable to expulsion from this society."

This resolution embraces what may be regarded as quackery. The use of secret remedies, all efforts to obtain business other than on fair and honorable terms of merit; all assumed excellence in operations; all puffing of self to the prejudice of the profession. Your reporter will find that the above resolution *was adopted,* and perhaps he would find enough which *had* been done to reflect on, without hunting up that which was not done, and using this as authority. But we find that the resolution which was offered at the same meeting of the society, which was "that any member of the society who may patent any instrument or improved mode of practice, may be subject to expulsion from the society." This resolution, which was first laid on the table, and next day withdrawn from the society by consent, and the gold medal resolution of the last meeting, which was also first thus withdrawn, and then referred to a committee, and in that committee also withdrawn. These resolutions offered, but not received or passed by the society, is of the utmost consequence, and shows the *exact views* of the association, so thinks your reporter. True, they do; without your reporter's *construction.* We refer him to the twenty-third chapter of Matthew, and twenty-third verse, and he will find that there was of old, a *certain class* which also omitted the "weightier matters of the law."

We have given, as we think, a fair and candid construction of the questions suggested; one which, as a profession, we may be able to abide by, and which cannot, in the least, effect its dignity and usefulness. Any other course would cut off from our intercourse, many of the most prominent in the profession, whose patents effect not, in the least, the curative treatment of dental disease, or any other, and who now offer the profession the use of their improvements on very reasonable terms.

We are free to confess we regard the disposition to patent every little improvement in instruments and appliances for use in mechanical dentistry, as illiberal, yet, so long as it is restricted within the bounds we have prescribed, we shall not complain. There are many improvements coming to us yearly, without any restrictions of the kind, and we are glad to think our dental periodicals are diffusing much useful knowledge which is free to all, and covered by no copy or patent right.

We are accused by your reporter, as having changed our views on this subject. And we perceive he has, with all the consequence of a village pettifogger, cross-examined us on the subject, and although he makes us say things we never thought of, still he is not able to make out a clear case. We did remark, however, that our views had, perhaps, undergone some change, yet not to that extent he would make the society believe, and referred him to the remarks of ours he had already quoted, to show that we had always thought "*a mechanical improvement might with propriety be patented*," and if we had changed at all, it was merely in the limits to which we would allow this to go, so far as the profession is concerned.

We did not, before or out of the society, contend or say "that Dr. Allen should be granted the right to patent," and the whole of your reporter's charge in the paragraph on page 247 of Journal, commencing with the above quotation, is incorrect, except one sentence, which is we regard the "improvement as an important one."

We remarked that we were not discussing the subject of patents—that we regarded it as not properly before the society, that the majority report did not recommend or say any thing of it, but merely reported on the merits of the improvement presented by Dr. Allen. Your reporter knows that the society had not time to enter into a discussion of this kind. Your reporter seems to think there is very little of dentistry, except that which is mechanical—that when this is gone, the "stock, lock, barrel, and even half of the flint is gone." Now, sir, we have always regarded this view as the greatest curse of our profession ; it is this which retards the progress of dental science. The great evil we are contending against in dental education, is this *idea*, that dentistry is a mere mechanical art. We know there are those in the profession, whose practice is mostly in this department, yet this does not do away with the necessity for a more enlarged mode or sphere of practice. And we know there are many in the profession who regard this as a mere appendage to surgical dentistry, and not half so important.

Your reporter, really, in his use of the Irishman and his Dutchman, (Steemer,) makes himself very amusing, and we only wonder that the society could retain its gravity at the exhibition which the Scotchman made of these very opposite characters. It so happens, that "we in Cincinnati," know something of the tryo, except the Irishman, and from the sample exhibited, we suppose he shot himself with the "half of a flint" which he found in his possession, and was duly buried by the others. We know not if the Dutchman has been able to advance any other in his ART, as your reporter *claims he has Dr. Allen.* We are told he made one other effort, and failed. From this, we may infer that Dr. Allen was a good and apt scholar, or the Dutchman has forgotten his method of teaching. We saw some specimens just before the meeting, which convinced us that the student had got in advance of his preceptor. "We in Cincinnati," we presume, embrace a few besides your reporter, if so, we dissent from him as to the time when Dr. Allen "first showed specimens of his work."

Your reporter mistakes, we did not *say* we were "shown the stethiscope," it was merely described to us.

Your reporter takes great credit for having, as he supposes, stopped the vote on Dr. Goddard's resolution. He says, "at this juncture, *I arose* and *arrested that act.*" Truly he makes a very Leonidas of himself, and how nobly he reports the battle. Drs. Goddard, Allen and Taylor's weapons fall as harmless as "snow flakes." How flat, tame and insipid their speeches appear, but the sword of Leonidas at every blow deals death and destruction. Tis true, Dr. Taylor knows how to second a resolution; this he does "pretty promptly," and then the President, "without a minute for discussion, was proceeding to take the vote." For shame, Mr. President—why such haste—did you not know that *Dr. Leslie* "is desirous to discuss the matter?" That he has a speech of full twenty pages to be delivered of, and also an *after birth* of two or three pages more. Now, sir, a man of your gravity and age, with over twenty years practice, should know that such cases cannot be postponed, at least longer than till "after dinner," even then we should take a "hasty plate of soup." We are rejoiced, Mr. President, to know that you had the humanity to wait on the gentleman. The dental precosity thus brought to light, might have died in *utero*, and the dental world been deprived of this wonder of the age.

But we seconded the gold medal resolution, and hence, *nolens volens*, must be for it; by the same rule, the minority report would have died in the hands of the committee. It happens, however, to be made of sterner stuff, and is harder to kill than the Irishman, for it cannot be *expunged.* We will here observe, that the mover of the expunging resolutions, designed to have it apply to the minority report, and every thing on the subject, but the report which was adopted as the sense of the association. We are, therefore, right, in not printing this report with our proceedings. It may have been handed to us with the other documents, if so, it passed with them into the fire, when the minutes were published. We offered, however, to publish the report, if your reporter would furnish a copy—he has not done so, and said it was of no consequence.

We are censured for not having shown the majority report before we had assembled in "the hall." Had your reporter met the committee in the morning, at the appointed time, he would have seen the report. As it was, he was in the same predicament with the majority of the committee. It appears that both had a report, and that your reporter did get to see that of the majority before we had reached the hall in which the society met. This is more than the majority can say of the minority, for we did not see his at all, until after it was presented to the society: of this, however, we do not complain. The majority of the committee had the right to present just such a report as they saw proper; and if they thought it best to retain the original preamble offered by Dr. Goddard, the society could accept or reject, as they saw best. The committee could approve of this without approving of the resolution which was attached.

We shall not, however, as we said at first, pretend to state all the misrepresentations of your reporter. We have merely glanced at a few of the prominent ones, and these generally as connected with ourself, and in doing this, we have seen fit, in some measure, to define our position in regard to patents. This we do cheerfully to the profession, and although we may differ in our opinions on this subject with some, yet it is frankly given, and we stand ever ready to pay due deference to the views of others. But we did not think the spirit manifested by your reporter before the society, required much at our hands.

We may be told, however, that the society evaded this question at its recent meeting. The fact is, this is not really true. The question was not considered as before the society at any time, and in no shape was it, but as dragged in by your reporter just before the society had determined to adjourn. The majority report had been accepted, and if not adopted, it was merely waiting the formality necessary for the minority report. The gold medal resolution was not acted upon or passed, because we thought it inconsistent to give a medal for that which the society did not receive. We wished to avoid the position of the American Society. We felt, however, that we could speak our minds freely in relation to what we considered an improvement in mechanical dentistry, and this we did as we think, without favor or prejudice. But, says your reporter, the society has changed its position on this subject, since its second annual meeting. This needs proof. The society then "postponed action" in relation to patents, and has ever since, your reporter's *speech* excepted. We might here assign, as a reason, (but we only name that which might be assigned,) that the society did not wish to stand alone on this subject; that it did not wish to part with several of its members, some of whom have patents, some of whom would like to have, and some of whom have tried. As the former, we may name, without being too particular, Dr. Allen; as the latter, *Dr. Leslie:* one the patentee of a plan for restoring the contour of the face, and also a new method of fastening teeth to plates. The other, as seeking a patent for *solder*. Yes, *solder*, yea, *solder*.

We have had a trip east described to us, for this ostensible purpose, and this patent should have been on the same shelf in the patent office, with the one which your reporter says, has "gone into oblivion." The patent office was visited, and the patent, *we* believe, not *obtained*, for what *reason* we know not, perhaps it wanted *originality.* It has been, however, suggested that the Dr. was driven to it by the meanness of others, and that he was afraid *we* would take the honors. O shades of dentistry, "*how our views have changed.*"

One more correction, and we are done.

Your reporter remarks, that "Dr. Taylor informed members of the society, that he would, during the coming year, conduct it (the Register) at his own expense, and with this understanding it was continued under his editorship." We utterly deny the whole of this charge. The whole of it is fabricated from this one statement we made, which was, that we had no doubt but that the Register would be able to meet its own expenses this year, but that we were re-elected editor with the understanding that we should pay the cost, provided the Register failed to meet it, is without truth. The fact is, the society stands in the same relation to the Register, as it always has. We have repeatedly solicited the society to relieve us of the editorship, and hence the inference of your reporter is not only unjust, but false, that some understanding of this kind, secured our re-election.

In conclusion, we would say to your reporter, that the *samples* we have recently seen in the "report," and by *letter*, to those high in the profession, gives us but a poor estimate of his capacities as a reporter. One essential element is wanting, which is *Truth.*

